# MODULATION OF MITOCHONDRIAL THIOREDOXIN IN AGE-RELATE MUSCLE ATROPHY AND INJURY

**DOI:** 10.1093/geroni/igad104.3479

**Published:** 2023-12-21

**Authors:** Morgan Barkley

**Affiliations:** University of Alabama at Birmingham, Birmingham, Alabama, United States

## Abstract

Maintenance of skeletal muscle is vital to health and wellbeing. Over the course of age there is a progressive decline in muscle mass and function. This decline results in frailty, reduced independence, reduced quality of life and elevated risk of mortality. Lean muscle mass contributes approximately 50% of body mass in a young adult while only 25% in an individual in their 80s. Critically, this decline in muscle mass is not continuous but composed of periods of stability punctuated by rapid declines. The chief driver of such events is loss due to injury related inactivity. Bed rest, is an essential part of recovering from injuries, however, long periods of inactivity can lead to skeletal muscle atrophy. A study of just 10 days bed rest in older adults produced a 6% decline in lower extremity lean muscle mass and a 15% decline in muscle function. Importantly much of the muscle mass lost even from short bed rest events are thought to never be recovered and to accelerate age related muscle declines. For this reason, development of interventions which reduce bed rest induced muscular atrophy would have considerable benefits in maintenance of independence for older adults. In my research project I am investigating a novel genetic target to ameliorate muscle atrophy as a consequence of immobility. We have found the protein TXNIP, a critical driver of apoptosis, to be induced under limb. Using TXNIP knockdown and TXNIP inhibitors I’m testing the hypothesis that prevention of TXNIP induction can reduce muscle atrophy.

